# Achieving the cancer moonshot in Africa

**DOI:** 10.3332/ecancer.2022.ed126

**Published:** 2022-11-10

**Authors:** Isaac Adewole, Emily Kobayashi, Megan O’Brien, Jackson Orem, Anne F Rositch, Wilfred Ngwa

**Affiliations:** 1 College of Medicine, University of Ibadan, Ibadan, Nigeria; 2 Clinton Health Access Initiative, Boston, MA, USA; 3 American Cancer Society, Washington, DC, USA; 4 Uganda Cancer Institute, Upper Mulago Hill Road, PO Box 3935, Kampala, Uganda; 5 Department of Epidemiology, Johns Hopkins Bloomberg School of Public Health, Baltimore, MD, USA; 6 Global Health Catalyst, Boston/Washington, USA; 7 Sidney Kimmel Comprehensive Cancer Center, School of Medicine, Johns Hopkins University, Baltimore, MD, USA

**Keywords:** cancer moonshot, africa, USA, global health, collaboration

## Abstract

For decades now, the United States (US) has been a leading contributor in global health with the government, academic institutions, foundations, non-profits and industry investing and partnering with African countries, as seen with the US President’s Emergency Plan for AIDS Relief (PEPFAR) program. Now as more people survive HIV/AIDs and other infectious diseases in Africa and live longer, non-communicable diseases like cancer are on the rise, in what can be described as a growing health iceberg, hidden under epidemics of infectious diseases. There is now more urgent need for international collaborations on cancer, which has become a leading cause of death in both Africa and the US, underpinned by poignant disparities in access to care. The re-ignited Cancer Moonshot in the USA and publication of the Lancet Oncology Commission report for sub-Saharan Africa in 2022 provide a timely and valuable framework for growing US-Africa collaborations in the coming years towards attaining the goal of the cancer moonshot both in the US and Africa. This goal is to reduce cancer death rate by at least 50% over the next 25 years, and to improve the experience of those living with and surviving cancer. The US-Africa summit taking place in Washington in December 2022 provides a momentous opportunity to identify recommendations or priority areas, some of them included in this article, and initiating action for win-win collaborations towards achieving the cancer moonshot in Africa.

## Introduction

In February 2022, United States (US) President Joe Biden launched the reignited cancer moonshot [1], setting a lofty goal of decreasing cancer deaths by at least 50% in the next 25 years and improving the experience of those living with and surviving cancer [1–5]. This ambitious initiative in collaboration with several others seeks to end cancer as we know it. Following that, the Lancet Oncology Commission (LOC) report for sub-Saharan Africa (SSA) [6] was also launched, highlighting a growing cancer crisis in SSA with a projected increase from 520,348 deaths in 2020 to about 1 million deaths per year by 2030. It included critical recommendations for achieving the UN Sustainable Development Goal to reduce premature mortality from non-communicable diseases like cancer by a third by 2030. The launch of the reignited moonshot, and the recommendations in the LOC report, point to a tremendous opportunity to strengthen US-Africa collaborations and finally begin to make a significant dent in cancer morbidity and mortality and improve quality of lives of cancer survivors. The reignited moonshot initiative comes with expressed commitment for global engagement including with Africa as highlighted during the launch of the LOC report for SSA [7].

Already the US government, academic institutions, foundations, non-profits and industry have been investing and partnering with African countries for global health. At the government level, we have seen programs in Africa like the US President’s Emergency Plan for AIDS Relief (PEPFAR), which along with US support in the Global Fund to Fight AIDS, Tuberculosis, and Malaria, have had a significant impact in Africa [8]. The US National Cancer Institute Center for global health has also been providing funding for research collaborations with African institutions in addition to initiatives involving US academic institutions, and non-profits [9]. However, given that cancer is now also a leading cause of death in Africa, with the situation expected to get worse, the goals of the cancer moonshot are equally or even more relevant to achieve in Africa.

## Ambitious but achievable goals

Much of the initial focus of the cancer moonshot in the US has been on development of cutting-edge interventions, some of which are in tandem with the Call to Action of the LOC. The LOC findings highlighted that there are opportunities to achieve moonshot goals in Africa by simply improving access to current effective interventions. To take one example, patients with breast cancer in Africa are half as likely to survive five years as those in the US – a disparity driven by late diagnosis, delays in care, and lack of access to basic surgery, medications, and radiotherapy treatments [6]. Despite increasing levels of commitment from African governments, there is still a substantial resource gap – one that the US could partner with African countries to help address with commitment from the reignited moonshot [10]. The LOC report for SSA includes examples of commitments from African governments, as seen with the example of Rwanda to eliminate cervical cancer and an increasing number of African governments developing National Cancer Control Plans that include government support for cancer care services [6].

However, more needs to be done urgently, including with specific targeted investments outside of research initiatives to help address gaps. Examples highlighted in the LOC report include: Investment in cancer diagnostics and imaging infrastructure. It has been shown that the benefits of scaling up diagnostic imaging for cancer in Africa would be substantial, with potential to avert more than 35% of total projected cancer deaths in Africa between 2020 and 2030 and result in over 60 million life-years saved [6]. Other areas that can be considered for greater investment include in pooled procurements as well as continuous negotiations with pharmaceutical companies and manufacturers of medical equipment to reduce the cost of cancer diagnosis and treatment. Investment in workforce training is also crucial to prevent millions of avoidable deaths by 2030 [6]. Meanwhile, Investing in universal health coverage to provide equity of access; investing in cancer registration to provide data upon which rational cancer planning will be undertaken; and investing in telehealth are also highlighted in the LOC calls for urgent action.

Potential sources of investments outside of government suggested in the LOC report include external sources such as the World Bank or African Development Bank, which may see providing funds as a good investment, or from other novel financing mechanisms such as diaspora bonds. Furthermore, it is possible to develop funding mechanisms where finances are mobilized, pooled, and then allocated to improve access to cancer medicines. This can draw on pre-existing examples as with the Global Fund to Fight AIDS, Tuberculosis, and Malaria, where there can be sustained impact in increasing access to care. The LOC report mentioned ‘access’ over 240 times, highlighting the opportunity to reduce mortality rates using already available approaches and technologies.

## Expanding already strong US commitment

There are areas where the US is already investing in SSA, for which there are opportunities to leverage existing resources and infrastructure for a more significant impact in reducing cancer mortality. One such priority area is in eliminating cervical cancers in SSA. According to the LOC report, cervical cancer is the leading cause of death in SSA. Still, it is the only cancer type that can be targeted for elimination with currently available prevention and treatment strategies. Some countries across Africa have already developed plans for achieving elimination, and begun rolling out the HPV vaccine and cervical cancer screening and treatment programs. The US Government has funded some of these programs, through robust support for Gavi, the Vaccine Alliance, and programs delivered through the nation’s aid agencies. However, in many places the full elimination strategies are not being implemented. Investments by the National Cancer Institute and other organizations have demonstrated feasible delivery models for cervical cancer screening and treatment services and could increase impact by: 1) expanding geographically within countries or to new countries, 2) expanding screening programs beyond women living with HIV, 3) incorporating breast cancer early detection (broader women’s health programs) and linkage to care, and/or 4) extending focus along the cancer cascade to strengthening treatment services for women with advanced cervical cancers. An extension of PEPFAR re-imagined to support elimination of cervical cancer would have a major impact, so that we do not save a life from HIV/AIDs only to lose it due to HIV-related cervical cancer.

In addition to building on the platform of PEPFAR, other approaches should be considered on how to expand existing platforms into the much-needed areas such as diagnostics and strategies for implementation of life saving surgery and other treatment modalities like chemotherapy and radiotherapy. Just like the US has leveraged PEPFAR to support the Global Fund to Fight HIV/AIDS, Tuberculosis, and Malaria, the reignited cancer moonshot initiative can be leveraged to catalyze set-up of a Global Fund to Fight Cancer. Also, in marshalling resources to fight cancer in Africa, the US African diaspora should be seriously considered as highlighted in the LOC for SSA. Considered the 6^th^ region of the African Union, the African diaspora has tremendous financial potential as seen with tens of billions of US dollars in remittances per year, at levels reported to outweigh foreign aid to the continent [11, 12]. We have highlighted policies and strategies for more effective engagement of the diaspora in addressing healthcare and cancer [13] that can be employed. Interestingly, one focus of the US-Africa leaders’ summit in December 2022 is to amplify diaspora ties [14]. This will provide an excellent platform for engaging the US Africa diaspora in supporting healthcare, and in particular addressing the growing cancer crises in Africa.

## US-Africa partnerships to learn from each other

Global health is local health; aid does not have to go one way only; bi-directional learning, as we close gaps in Africa, could also provide opportunities to stimulate further collaborations or innovations and address disparities in the US. While the US leads the world in available innovative therapies, broad and equal access is also a significant issue, with disparities in access to care among the greatest in the world. For example, an analysis of American Cancer Society reports[15, 16] highlights grievous cancer health inequalities, with the highest cancer mortality rates for African American.[17]. These disparities are associated with barriers to access to cancer control interventions, such as geographic, costs/poverty, and cultural barriers that can be addressed. For example, during the COVID-19 pandemic, healthcare professionals had to innovate in both the US and Africa to increase access. Examples of approaches taken include the initiation of breast cancer patients on oral medicines as they awaited surgery and chemotherapy dates, use of telehealth and hypofractionated radiotherapy highlighted in the LOC, which could address disparities in access both in the US and Africa.

## Aligning the cancer moonshot and LOC for SSA

**Figure 1.**
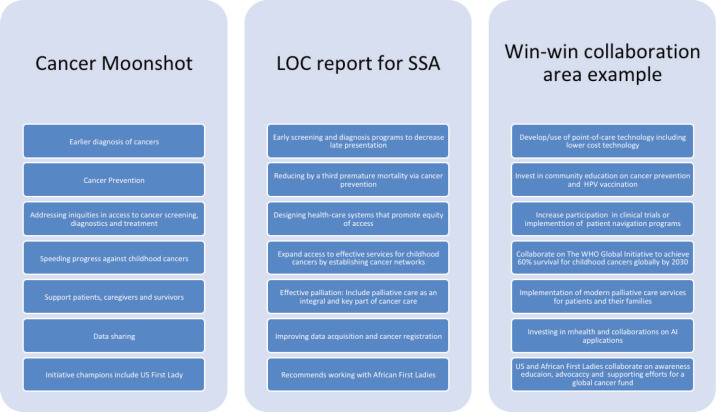
Potential areas and opportunities where the Cancer Moonshot aligns with the LOC priorities for sub-Saharan Africa for win-win collaborations.

In developing a plan for “Global health is Local Health” or win-win partnerships, we must consider how to align the reignited cancer moonshot priorities [3] with specific opportunities to improve access to care in SSA, as highlighted in the LOC report. Here win-win partnerships refer to collaboration opportunities that can benefit all partners. Growing such partnerships could help towards achieving the cancer moonshot goals both in the USA and Africa. Figure 1 highlights some of these potential opportunities for alignment.

The US previously committed to a bilateral effort with the United Kingdom (UK) to take on the challenges of cancer together [1], and a US-UK scientific meeting resulted in recommendations on how the two nations can collaborate. A similar initiative involving the US and African leaders, potentially as part of the healthcare agenda during the US-African Leaders summit in Washington December 13-15, 2022, could identify recommendations for US-Africa collaboration. There may be opportunity for First Lady Dr Jill Biden to work with the African First Ladies, many of whom have great interest in leading the fight against women’s and childhood cancers in their countries [18, 19]. The African diaspora including African Ambassadors could be major catalysts in growing US-Africa collaborations towards this shared goal of curbing the growing cancer burden and disparities [6].

## Conclusion

In conclusion, as African leaders travel to Washington in December, the growing cancer crises in Africa should be a priority, especially as President Biden also considers cancer a priority for the US. As the US and Africa consider bilateral collaborations in healthcare, there is a tremendous opportunity to develop a strategy that aligns the reignited cancer moonshot priorities with those described in the LOC for SSA. Investing in win-win partnerships around increasing access could make the cancer moonshot achievable in Africa, perhaps even more readily, while also contributing to reducing disparities in the US. The vision to extend the cancer moonshot globally could even catalyze US leadership in mobilizing further international support, including via a new Global Fund to Fight Cancer, which can catalyze international action to end cancer as we know it.

## Conflicts of interest

The authors declare there is no conflict of interest.

## Financial conflicts of interest

The authors declare there are no financial conflicts of interest.

## References

[1] The White House (2022). Fact sheet: President Biden reignites cancer moonshot to end cancer as we know it. https://www.whitehouse.gov/briefing-room/statements-releases/2022/02/02/fact-sheet-president-biden-reignites-cancer-moonshot-to-end-cancer-as-weknow-it/.

[2] Agus DB, Jaffee EM, Van Dang C (2021). Cancer Moonshot 2.0. Lancet Oncol.

[3] Gourd E (2022). President Biden outlines plans for Cancer Moonshot 2.0. Lancet Oncol.

[4] Ledford H (2022). Cancer ‘moonshot’ has lofty new goal: halve deaths in 25 years. Nature.

[5] Singer DS (2022). A new phase of the Cancer Moonshot to end cancer as we know it. Nat Med.

[6] Ngwa W, Addai BW, Adewole I (2022). Cancer in sub-Saharan Africa: a Lancet Oncology Commission. Lancet Oncol.

[7] Global-Health-Catalyst-Summit (2022). Global health catalyst summit. https://www.globalhealthcatalystsummit.org/.

[8] Fauci AS, Lane HC (2020). Four decades of HIV/AIDS – much accomplished, much to do. N Engl J Med.

[9] Strotherab RM, Asirwa FC, Busakhala NB (2013). AMPATH-oncology: a model for comprehensive cancer care in sub-Saharan Africa. J Cancer Policy.

[10] Cabanes A, O’Brien M (2021). It’s Time for a New US Cancer Initiative in Africa.

[11] Doyle M (2013). Africans’ remittances outweigh Western aid. https://www.bbc.com/news/world-africa-22169474.

[12] Ngwa W, Ngoma T (2016). ed W Ngwa (Bristol: IOP Publishing). Emerging Models for Global Health in Radiation Oncology.

[13] Elzawawy A, Ngwa W (2022). ed W Ngwa (Bristol: IOP Publishing). Approaching Global Oncology: The Win-Win Model.

[14] U.S.-Department-of-State (2022). U.S.-Africa leaders summit. https://www.state.gov/africasummit/.

[15] American Cancer Society (2022). Advancing health equity – addressing cancer disparities.

[16] Sengupta R, Honey K (2020). AACR cancer disparities progress report 2020: achieving the bold vision of health equity for racial and ethnic minorities and other underserved populations. Cancer Epidemiol Biomarkers Prev.

[17] Beavis AL, Gravitt PE, Rositch AF (2017). Hysterectomy-corrected cervical cancer mortality rates reveal a larger racial disparity in the United States. Cancer.

[18] Elrefaei A (2019). IAEA Promotes Closer Cooperation at the First Ladies International Symposium on the Burden of Cancer in Africa.

[19] Oluwole D (2013). African first ladies and the fight against women’s cancers: iron sharpening iron. https://www.bushcenter.org/publications/articles/2013/02/african-first-ladies-and-the-fight-against-womens-cancers-iron-sharpening-iron.html.

